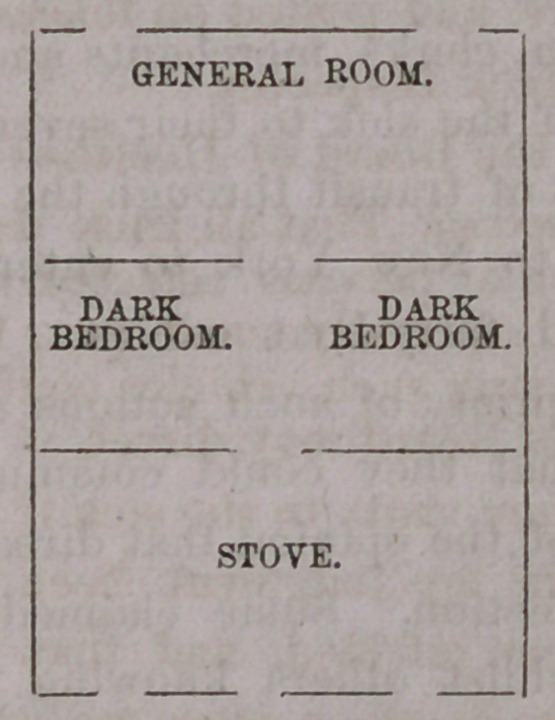# Transactions of the Medical Society of the County of Kings

**Published:** 1865-12

**Authors:** J. B. Jones


BUFFALO

Medical and Surgical Journal.

VOL. V. DECEMBER, 1865. No. 5.

ART. I.—*Transactions of the Medical Society of the County of Kings.*

REGULAR MEETING, SEPTEMBER, 1865.

*Small-Pox in Cities—The causes of its spreading and the means essential for checking it.* By J. B. Jones, M. D.

It would have been presumption on my part to have taxed the time of the members of this Society by presenting a paper on variolus disease, containing my observations and the deductions or conclusions therefrom derived from my researches or practice, as an individual private practitioner, especially so when it will be borne in mind that we have had several valuable and apparently exhaustive papers, but recently presented on the same subject by other members of this Society, which papers will ever stand as monuments of industry and research, and stamp their authors as true lovers of science and ornaments to the medical profession.

But, Sir, occupying the position of health officer of the city, I, under our laws, become possessed of a vast amount of material facts not possible to be acquired by the entire corps of practitioners in the city. The papers referred to generalize, while the facts I present are local, and pertain chiefly to Brooklyn, and affect us all both socially and professionally. They corroborate many of the statements contained in the papers of Drs. Bell, Hutchison and others, and are of value on that account, if no other. They may serve to strengthen opinions deduced from individual observation in private practice, and may perchance enlighten some, and be the means of removing prejudices now known to exist in the profession in relation to actions necessary to be taken municipal, professional, and by the community, when the disease is present, and especially what actions are imperatively demanded to *prevent* its presence as far as lays in our conjoint power.

The health laws of Brooklyn require every person practicing physic in the city, who has any patient sick of a malignant, contagious, infectious, or pestilential disease, to make and file in the office of the Board of Health, within such time as the said Board may determine, the name of such patient, the house and place, and the name and nature of such disease, to the best of his knowledge and belief, failing to do which he is subject to a fine of $250, or six months imprisonment, one, or both. (See Title IX, City Charter.)

This law, without material alteration, has existed since Brooklyn was a village, and has been the source of much just complaint from physicians, for were they to observe it to the letter a large portion of their time would be occupied in their visits to the Board of Health to make unnecessary, useless reports, and in not a few instances it would require them to expose matters confidentially entrusted to them, the keeping of which they are professionally and in honor bound to do, and are sustained by the courts in so doing.

This matter I called the attention of the Board of Health to, and was requested by that body to draft such a law as would in my judgment attain the object desired, and relieve the physicians from unnecessary trouble. This was done, presented to the Board, approved of by them, and a resolution passed recommending our representatives to secure its passage. Previous to its presentation it was submitted to a number of distinguished members of the profession, and met with their entire approval. The following is a copy of the proposed law:

"Every person practicing physic in the said city, who shall have a patient sick of small-pox, yellow, typhus, typhoid or spotted fever, or cholera, shall make and file a written certificate thereof within twenty-four hours after his first attendance on, or the earliest development of such disease, in such patient, stating the name, age, nativity, disease and house, or place where such patient then shall be, in the office of the Board of Health.

And the Board of Heath may require every and any person practicing physic in said city who shall have any patient sick of the above mentioned or any other contagious, infectious, malignant or pestilential disease, in any manner susceptible or capable of being controlled or influenced by the amount of general or local causation, or whose virulence or spread are in any manner capable of being controlled or prevented by public or private sanitary regulations or hygienic measures, or where by the adoption of such regulations or measures the community can be protected or benefited, to make and file in said office within such time as they may prescribe, not less than three hours after service of a copy thereof upon him, an affidavit stating therein whether he has or has not any patient, who in his opinion shall then be sick of any such disease, and if he has any such patient to state in such affidavit his or her name, and the house or place in said city where he or she shall then be, and the name or nature of such disease, to the best of his knowledge and belief."

The advantages of such a law are so apparent that argument is unnecessary. The only argument, if such it could be called, against a law of that character, that has been presented to me was to the effect that the authorities had no right to tax a physician's time without compensation. In place of its being a tax on the time of medical men without compensation, it might rather be designated an attempt to prevent the improper use of their time whereby they derived unlawful compensation, for what right, moral or constitutional, has any man to cause the spread of disease, or to act or dispose of his time so as to deteriorate health and shorten life because he finds it profitable to do so?

When these returns are made they are placed on record. It is the duty of the health officer to visit *all* such cases, examine them and their surroundings, and report in writing to the Board of Health his opinion of the nature of their sickness, surroundings, etc., whereupon said board take action.

If the sick are so circumstanced that they can be completely isolated, properly nursed and otherwise attended, having nourishment and properly ventilated apartments, and are not in crowded tenements, they are left at their homes, if they are not thus circumstanced they are removed in a vehicle especially provided and set apart for that purpose to the hospital, provided their lives are in no wise jeopardized thereby; if they are too sick to be removed, they are supplied with all things necessary, such as medical attendance, medicine, nurses, food, etc.; in either case, wealthy or poor, when left at their homes, communication with them except by physicians, nurses, and persons conveying them food, is prevented. No articles are allowed to be removed from the sick chamber that might convey the infection to others. When the patient has convalesced all such goods are taken charge of by the authorities, and the proper cleansing of the premises are secured under their supervision or direction.

When the sick are removed to the hospital, if able, they must pay expenses incurred on their account, by the authorities, including board at the hospital; if they are not able to pay, the city "foots" the bill.

It is very gratifying to be able to state that a number of wealthy persons, effected with variolus disease, availed themselves of the advantages of our hospital during their sickness, and subsequently expressed themselves as feeling grateful for the careful nursing, fine accommodations, medical and general attendance they received. Such testimony from persons who have luxurious homes will lead you to anticipate the laudatory testimony of the poorer classes who occupied adjacent beds, particularly when the fact is known that the poorest patient receives the same care, attention, etc., in common with the wealthiest; the poor laborer with the rich merchant occupy the same ward, lying side by side, attended by the same physician and nurse, and subject to the same rules, etc.

When the sick are removed, all infected articles in the apartments they are taken from, are seized by the authorities, taken to a proper place and destroyed by burning them. If the goods thus destroyed belong to persons who will not suffer by their loss, no restitution is made, but if they belong to persons who are poor, and they constitute the majority, and who would seriously suffer by such loss, the Board of Health make restitution to such extent as in their opinion the case demands.

The owners, agents and occupants of premises where such sick are located or removed from at a proper time are notified to cleanse, purify, disinfect, etc., such premises within a prescribed time; failing to do which the authorities cause the same to be done at the expense of such owner, etc. In removing the sick the less frequented streets and roads are driven through, and the patient closely covered.

Visitors are not permitted to enter the hospital where such persons are taken, except when a fatal issue is anticipated, then a relative or friend is admitted, and when so admitted they must not leave until the sick is either convalescent or has ceased to live, and in either case not until such measures are adopted that the community are secured from danger by contact with them. All persons interested in the welfare of the sick can send things to and commnnicate with them through the health department daily, which is distant from the hospital about 3½ miles.

I was enabled from former experience in this department to anticipate the presence of this disease in our midst, and early in the year made preparations accordingly. The Board of Health were convened a month earlier than ususual, public attention called to the subject, a large supply of most excellent lymph procured, etc. In May, thirty-two cases were reported, at which time active measures were adopted, but in August they were temporarily suspended, only six cases having been reported, and the fund of the Board having become exhausted, the Board deemed it best to await further developments before applying to the Common Council for a special fund to meet the emergency. The disease gradually increased from this time. In November it was deemed necessary to raise the monies necessary to carry out vigorous measures. The President of the Kings County Medical Society was invited to meet the authorities in consultation, at which meeting the means and measures necessary agreed upon, the Common Council concurring in the opinions expressed by the Board of Health, promptly appropriated the amount asked for.

Physicians were employed in each ward to visit every house and proffer vaccination, free of charge, to all. If several cases occurred in any one neighborhood, we immediately surrounded such vicinity by a cordon of physicians who visited every person in such district, and vaccinated as speedily as possible all who could be prevailed upon to accept the boon.

The Board of Education were notified of the presence of the disease and arrangements made with them to have physicians attend daily at the various schools to perform vaccination and revaccination upon all who would then accept. The Board at once put in force the law of 1860, a special act pertaining to the public schools of Brooklyn, which states that no child shall attend the public schools of this city unless they are or have been vaccinated. This law almost makes vaccination of school children compulsory.

The proprietors of factories, the principals of academies, and the presidents of companies where numbers of persons daily congregated, were notified of the existence of the disease, proper sanitary regulations suggested to them, and the fact made known that any one could be vaccinated without charge at their homes, or at the several dispensaries throughout the city where provisions had been made to that end.

Railroad and ferry companies were strictly enjoined from carrying passengers affected with this disease.

The following resolution was printed and sent to the livery stable keepers and hackmen in the city, advertised in the corporation papers, and posted on fences about the city, under the direction of the Hack Inspector.

In the Board of Health—

*Resolved*, That all hack drivers are hereby forbid carrying any person or persons infected with any contagious or infectious disease, especially small pox, in their hacks, etc., under the penalty of having such vehicles confiscated and destroyed, in such manner as this Board may direct.

In my visits to the sick if I found that any member of the family had in my judgment been exposed and his person or clothing become infected, and that he or they worked in any store, shop, factory or other place where others could, through him or them, be infected, information was immediately furnished the proprietor, foreman or other person having charge of such places.

Superintendents of Sunday Schools were duly informed of cases occurring in the families of the attendants of their respective schools. We have gone into week-day and Sunday schools, and taken therefrom children who had gone thereto direct from an infected place; in some instances direct from personal contact with infected persons. In several instances small-pox matter has been found adhering to their clothes.

Wherever a case occurred, if there were more than one family in the house, all were notified, the residents adjoining, and those residing in the second and third premises therefrom were advised of its presence in the vicinity, vaccination and re-vaccination recommended, and in many instances at once performed.

We have in this city a number of small retail stores, many, and I think the majority of the owners thereof, with their families, large or small, as the case may be, rent and occupy but the one floor; this floor they divide into several apartments by means of wooden partitions, which they generally cover with paper. These partitions as a general thing reach from half to two-thirds the distance from the floor to the ceiling. In front they keep their goods for sale. Next to this place they devote a small place for sleeping purposes, and to the rear of the sleeping room the space is used for kitchen, dining, sitting and general living room. In many cases there is but one partition, with the store on one side, and the sleeping, cooking and all other matters incident to family affairs transacted on the other side. The diagrams will illustrate manner of dividing the apartment:

In the beds I have found as many as three cases of small-pox; in one instance I found three in one bed sick with small-pox, and in the general living room one dead of the same disease, several of the neighbors sitting therein, and the family dining in the store. In passing from the general room to the store you came in direct contact with the sick, the wooden partitions reaching only half or two-thirds of the way to the ceilings, one atmosphere was common to all apartments on that floor.

In such pest places, friends, neighbors and customers were received, entertained and waited upon, by persons in many instances direct from the bedside of the sick or dead. I have even found children sick with this disease lying under a counter, over which the mother was selling goods to a customer.

These citations will serve as types of hundreds of others met with during the prevalence of this disease. In all such cases I caused the stores to be closed, and adopted other effectual measures to protect the public.

In many places I found from one to six dozen each, of pants, vests, and coats in the hands of workmen to be made up, principally for the large clothing houses of the city of New York. These goods were designed not only for the civilian, but in part for the use of our brave soldiers. I have seen such goods used as pillows by persons covered with small-pox. I have found them in cribs and in beds, serving as quilts to cover the sick, and upon examination have, in a number of instances, found small-pox matter deposited on such goods. All such goods found have been seized and burned.

I have known coachmen, car conductors and drivers, washer-women, clerks, merchants and laborers, to go direct from the bed-side of the sick to their several vocations, using the city cars as a means of transit through the city, and those whose business called them to New York to enter the densely and very improperly packed ferry-boat cabins. When remonstrated with upon the impropriety of such actions some professed to be ignorant of the fact that they could communicate the disease to others. Some were of the opinion that direct contact with the sick was essential for infection. Some claimed that they were necessitated thus to act, whilst others knowing that they could spread the disease seemed totally indifferent whether they did or not. Remedial measures were adopted to meet all such cases.

It is a common practice to keep the soiled linen from the person and bed, as the changes are made for a week in the room with the sick, and then deliver such clothes to some wash-woman to be taken to her home to be cleaned. One family who were very much afraid of the disease spreading among the other members thereof, collected them as stated, and when the washer-woman called for them they would be thrown to her from the window of the sick room to the sidewalk! This wash-woman took the clothes to her residence, a tenement-house in which resided over one hundred persons.

It is not uncommon to find cases of small-pox in the same room where clothes are being washed and ironed, the nurse being the washer-woman, going from the bed-side to the tub or ironing-table, or from an infected nursing child she may have just had in her arms direct to her patrons, with these undoubtedly infected clothes. The amount of abject poverty discovered, distress relieved, and good accomplished, other than that which relates to small-pox by these visits of inspection would undoubtedly be highly interesting and instructive, but they do not pertain to the subject under consideration. I therefore reluctantly omit them. The beneficial effect of these combined measures have in each instance of the appearance of the disease in this city been markedly apparent. The brief histories of the following cases will afford the members of the Society material upon which to base their actions in the event of cases of small-pox occurring in their practice:*

Of the 818 cases of variola, reported as such, I found 216 with evidences of having been vaccinated, 13 of the number could not show any cicatrix, but had one or more members of the family to bear witness to the fact that in early life they had been vaccinated. Twelve additional cases were found who had been repeatedly vaccinated without effect. Sixty-two stated that they had been vaccinated in infancy and felt secure, but they had no evidence of the fact either upon their own persons or from the testimony of others.

In three cases other than the above, reported as variola, the patients were found to have well marked scarlatina anginosa. Cases were visited on the 4th and again on the 10th day of the disease. In seven cases *well marked* measles were found instead of variola.

In two cases, other than the above, an eruption was found confined to the scalp, one proved to be porrigo and the other impetigo.

In 558 cases reported as variola there were found 17 cases of confluent variola, and 71 variola discuta.

In 37 cases, other than the above, reported as varioloid, 19 different diseases were found.

The rate of deaths from variola during 1864 was $19\frac{23}{169}$ in 100, or nearly 1 in 34.

According to my records the small-pox is most prevalent under one year of age, 132 cases occurring, from one to five years only

* The cases referred to illustrative of the foregoing remarks are here omitted.

60 cases occurred. From five to twenty years of age there occurred 449 cases. From twenty to thirty there occurred only 148 cases, while from thirty to forty 106, and after that up to fifty 32 cases.

According to the same records varioloid is less prevalent under one year and most prevalent between five and ten years; in the former 17 cases were r eported; in the latter 63. Varioloid gradually declines from twenty.

The number of persons vaccinated was 36,690, of which 8,547 were primary, 16,335 were re-vaccinations, and 11,808 were returned as vaccinated, without designating whether they were primary or re-vaccinations.

Twenty-seven physicians were employed to perform this work, whose aggregate time amounted to 1029 days. They received for their services $4,116, making an average cost for each person vaccinated of about 12 cents!

Can anything more be done for Brooklyn in relation to this small-pox question? I answer emphatically, yes, not only for Brooklyn, but for the entire country. Give us laws making it compulsory for every one born in the State, or resident thereof, to be vaccinated and re-vaccinated; economy, justice and humanity demand it.

We may not by such laws be able to exterminate the disease, but we can so protect the people that the mortality will be diminished to such an extent that death from it would be rare and cease to excite the public mind.

From observation and research I am led to strenuously recommend re-vaccination to every person under thirty years of age as often as every fifth or sixth year. That small-pox is at all times present to a greater or less extent in all large metropolitan, commercial cities and adjacent places is beyond dispute.

That it cannot exist in the first mentioned places, in any considerable numbers without spreading to those places that are connected therewith by the ordinary means of travel, even should such places be distant hundreds of miles therefrom, has been made evident and placed beyond cavil.

That those places immediately bordering great cities are the more seriously effected is a logical deduction. That the means at present used cannot prevent its introduction into or its appearance in such places is made patent by experience.

That the laws in this State, and I believe in every other State in the Union, are culpably deficient, must be evident to the casual observer. That by judicious laws properly applied in cities and throughout the States, the number of *cases* which would occur annually, would not amount in the aggregate to the number of *deaths* which now annually result from this disease. That the reason why such laws are not now existing in this country, is due to a very great extent to the want of intelligent and combined action of the medical profession.

## Figures and Tables

**Figure f1:**
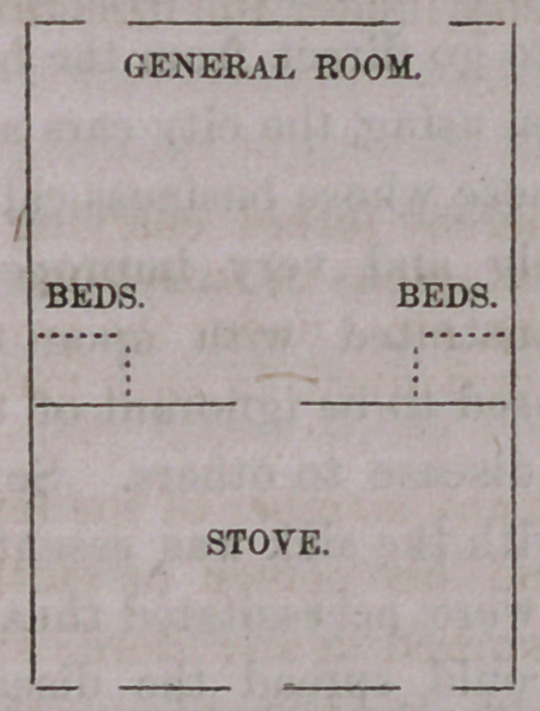


**Figure f2:**